# Molecular Characterization of the Circulating Strains of *Vibrio cholerae* during 2010 Cholera Outbreak in Nigeria

**DOI:** 10.3329/jhpn.v31i2.16381

**Published:** 2013-06

**Authors:** Kolawole S. Oyedeji, Mary-Theresa Niemogha, Francisca O. Nwaokorie, Tajudeen A. Bamidele, Michael Ochoga, Kehinde A. Akinsinde, Bartholomew I. Brai, David Oladele, Emmanuel A. Omonigbehin, Moses Bamidele, Toun W. Fesobi, Adesola Z. Musa, Adeniyi K. Adeneye, Stella I. Smith, Innocent A. Ujah

**Affiliations:** ^1^Molecular Biology and Biotechnology Division, Nigerian Institute of Medical Research, Yaba, Lagos;; ^2^Federal Ministry of Health, Abuja, Nigeria;; ^3^Clinical Sciences Division,; ^4^Monitoring and Evaluation Unit,; ^5^Public Health Division,; ^6^Administration Division, Nigerian Institute of Medical Research, Yaba, Lagos

**Keywords:** Cholera toxin gene A, Classical, Ogawa, *V. cholerae*, Nigeria

## Abstract

This study aimed at characterizing the phenotypic and toxigenic status of circulating strains of cholera during outbreaks in Nigeria, employing molecular typing techniques. Two hundred and one samples of rectal swabs, stool, vomitus, water (from the well, borehole, sachet, stream, and tap) and disinfectants (sodium hypochlorite) were collected from three states in the country. The samples were inoculated on thiosulphate-citrate bile salt-sucrose (TCBS), Cary-Blair transport medium and smeared on glass slides for direct examination. The *Vibrio cholerae* isolates were serotyped, biotyped, and characterized using PCR of the cytotoxin gene A (*ctx*A), *wbe*O1, and *wbf*O139 gene primer. Of the 201 samples screened, 96 were positive for *V. cholerae* O1 (48%), with 69 (72%) positive for *ctx*A gene. The results from this study showed that the circulating strains of cholera in Nigeria were of Ogawa serotype, also observed in other outbreaks in Nigeria (1991, 1992, and 1996). However, the strains were of the Classical biotype and were mainly (72%) *ctx*A gene-positive. This current investigation has confirmed the production of cholera toxin by the circulating strains, and this could be harnessed for possible cholera vaccine production in Nigeria.

## INTRODUCTION

*Vibrio cholerae* is the causative agent of cholera. It is acquired by consuming food or water contaminated with *V. cholerae*; strains belonging to O1 and O139 serogroups are agents of endemic and pandemic cholera, a potentially life-threatening diarrhoea that produces characteristic rice-water stool. It is a major problem in the developing countries and has been linked to poverty and poor sanitation (1,2).

Epidemiologically, cholera most often occurs in explosive outbreaks throughout several regions simultaneously. Pandemics of cholera have followed progressive patterns, affecting many countries across the continent and over many years (3,4).

Epidemic *V. cholerae* are classified into two serogroups O1 and O139 as well as two biotypes El Tor and Classical. The ongoing seventh pandemic is due *to V. cholerae* serogroup O1 biotype El Tor. There are also two serotypes of epidemic cholera, Ogawa and Inaba (5,6).

Between 1995 and 2005, a total of 632 cholera outbreaks were reported worldwide; 66.0% of the total cases and 87.6% of fatal cases were reported from sub-Saharan Africa (7). The World Health Organization (WHO) annual figures on global cholera incidence, which are based on official cases reported by affected countries, are believed to be under-estimated due to limitations relating to a lack of adequate surveillance systems (8). In addition, the actual number of cholera cases globally is estimated to be much higher than officially reported (8) because outbreaks are often not reported to avoid the risk of travel and trade embargos on the affected country. According to WHO, only five countries (Burundi, Cameroon, the Democratic Republic of Congo [DRC], Ghana, and Tanzania) have reported cases of cholera every year since 1990 (9). Prompt and accurate diagnosis of *V. cholerae*, therefore, remains a key step in cholera outbreak surveillance, which can greatly influence rapid intervention and prevention to minimize spread of disease and mortality.

Due to inadequate disease notification system in the country, it is believed that most cases of cholera are not reported due to poor surveillance systems. Fatality rates are 5% of total cases in Africa and less than 1% elsewhere (10).

Cholera exists as a seasonal disease in Nigeria, occurring annually mostly during rainy seasons. In most of the affected areas, cholera looks endemic since the cases occur round the year but with different magnitudes. It may occur in southern part of the state and later shift to the northern part. The disease is more pronounced in the border areas of the affected states, and the reason is attributed to migration of the population and personal hygiene of the affected people.

In Nigeria, the Federal Ministry of Health reported 37,289 cases and 1,434 deaths between January and October 2010 (11). Reported cases almost tripled compared to those of 2009, and 80% were women and children.

In 2010, cholera outbreak had been more widespread and deadly than in almost 20 years (11). The cholera epidemic was first reported at Taraba state, and it quickly spread to 17 more states, including some states in the southern part of Nigeria.

In a separate report by Federal Ministry of Health, it was shown that, in 2011 alone (till 18 November 2011), a total of 22,797 cases of cholera with 728 deaths and case-fatality rate (CFR) 3.2% has been recorded (12). This is, however, not as high as the recorded cholera cases in 2010. Hence, WHO described the 2010 cholera outbreak as the worst in Nigeria since 1991 when 7,654 people died of cholera out of 59,478 cases (13).

The toxigenic strains of *V. cholerae* produce the strains of *V. cholerae* carrying the cholera toxin gene (*ctx*) and have the potential to produce cholera toxin (CT). These toxigenic strains are responsible for cholera epidemics. Water is recognized as the most important vehicle for cholera transmission (2).

In Africa and, indeed, Nigeria which is one of the most-hit countries, the cause of cholera has been attributed to heavy seasonal rainfall and poor sanitation.

During the 2010 cholera outbreak in Nigeria, Nigerian Institute of Medical Research Emergency Response Team (NIMRERT) was deployed in some affected states, specifically Borno, Bauchi and Gombe states with the aim of isolating and characterizing the circulating causative agents during outbreaks, using serologic characterization, biotyping, and PCR analysis of the *ctx*A, *wbe*O1, and *wbf*O139 genes.

Ethical approval was obtained from the Institutional Review Board (NIMR-IRB) of Nigerian Institute of Medical Research before the start of the project.

## MATERIALS AND METHODS

### Sample collection

Rectal swabs were collected from the in-patients in the various treatment centres and/or camps visited and then inoculated as specified below. Three rectal swabs were collected from each participant or legal representative who consented during the outbreak, with the aid of the sterile swab sticks.

In total, 201 samples were collected from the three states visited—Borno, Gombe, and Bauchi—comprising 171 rectal swabs, 20 water samples (i.e. six from well, three from borehole, four from sachet, two from stream, one from tap, and four disinfectant solutions), 5 stool samples, 5 vomitus samples (from some patients among the 171). One of the swabs was used in inoculating the Cary-Blair transport medium, the other streaked directly on the thiosulphate-citrate bile salts-sucrose agar (TCBS), and the third smeared directly on slide for Gram-staining. Other samples, such as vomitus, water, sewage, and disinfectants (sodium hypochlorite) were collected and inoculated in alkaline peptone water and incubated for further processing. The water samples were collected randomly from the drinking-water sources of the participants, such as well, borehole, sachet, stream, and tap while the vomitus samples were collected from some of the in-patients. The disinfectants used in the treatment sites were also sampled to rule out contamination while the sewage samples were randomly selected based on information given by the participants in the questionnaire.

The samples were further processed on arrival at the laboratory.

It is pertinent to mention that the samples were collected from new cases without antibiotics and some prolonged admission cases that had been on antibiotics.

### Laboratory diagnosis

#### Isolation and confirmation of V. cholerae strains

Samples transported in Cary-Blair medium were separately inoculated into alkaline peptone water (pH 8.6) for growth enrichment at 37 ^o^C for 24 h. Each bacterial culture was then subcultured onto TCBS agar at 37 ^0^C for 24 h. Suspected *Vibrio cholerae* colonies were picked and subcultured for purity in a new TCBS plate called the purity plate. Each pure isolate of *V. cholerae* was serotyped for Ogawa and Inaba classification by slide agglutination, using serotype-specific antisera (Biorad, USA). Serogrouping of the isolates, based on the use of serogroup O1 and O139-specific antisera, was done using the slide-agglutination technique. Isolates other than suspected *V. cholerae* were also identified after preparing purity plates on MacConkey agar, using standard methods.

#### Characterization of the isolated strains

The isolated organisms were identified by growth of yellow colonies on TCBS agar and production of purple colour on reaction with oxidase reagent. The isolates were tested for lactose and glucose fermentation, using Kligers Iron agar (KIA), motility, and indole and urease production (MIU). Methyl Red test and String test were also performed.

#### Storage of strains

Each isolate was stored in a cryovial containing glycerol (20%) tryptone soy broth at −20 ^0^C.

#### Serologic characterization

Suspected colonies from TCBS agar were serogrouped with O1 and O139-specific antisera by slide agglutination. These were then serotyped with Ogawa and Inaba antisera (BioRad, USA).

#### Antibiotics susceptibility testing

The *V. cholerae* isolates were subcultured onto Mueller Hinton agar and their susceptibility patterns determined following procedure as described by CLSI (14). The antibiotics used were: augmentin, ofloxacin, gentamicin, nalidixic acid, nitrofurantoin, cotrimoxazole, amoxicillin, tetracycline, and doxycycline.

#### Genomic DNA preparation

Genomic DNA of each isolate was prepared by overnight boiling of broth culture and purification of the isolated DNA.

#### Amplification of the cytotoxin (ctxA), wbeO1 and wbfO139 genes

The *ctx*A gene VCT1 [5’-CTCAGACGGGATTTGTTAGGCACG-3’], sense strand and VCT2 [5’-TCTATCTCTGTAGCCCCTATTACG-3’], antisense strand, the *wbe*O1 gene : O1 *wbe*F [5’- GTTTCACTGAACAGATGGG-3’], O1 *wbe*R [5’-GGTCATCTGTAAGTACAAC-3’] and wbfO139 gene O139F2 [5’-AGCCTCTTTATTACGGGTGG-3’], sense strand and O139R2 [5’-GTCAAACCCGATCGTAAAGG-3’] from each isolate were amplified by PCR with 50-200 ng of DNA mixed with PCR buffer (20 mM Tris-HCl, pH 8.3; 50 mM KCl) containing 200 micromole each of dNTP, 1.5 mM MgCl_2_, 30 picomole each of forward and reverse primer, and 1 unit of Taq polymerase. The reaction was conducted in a thermocycler (Eppendorf Mastercycler pro, Hamburg), with an initial denaturation at 95 ^0^C for 5 min. This was followed by 35 rounds of amplification defined by denaturation at 95 ^0^C for 1 min, primer annealing done at 60 ^0^C for 1 min for the *ctx*A gene and the *wbe*O1 gene while the annealing temperature for the *wbf*O139 gene was 55 °C for 1 min. The primer extension for all three genes was at 72 °C for 1 min. A final extension step at 72 °C for 5 min was done, and the PCR product was kept at 4 °C prior to analysis.

#### Agarose gel electrophoresis

The PCR products were electrophoresed horizontally on 1.5% agarose gel that was prestained with ethidium bromide at 100 volt for 45 min, and 100 bp ladder (Promega, USA) was used as DNA molecular weight standard. The amplicon-sizes were visualized under UV light for *ctx*A gene, *wbe*O1, and *wbf* O139 genes to be 302 bp, 192 bp, and 449 bp respectively. Photo documentation was done with the aid of a photo-documentation system by CLINX Science Instruments, China.

## RESULTS

In total, 201 samples were collected from the three states visited—Borno, Gombe, and Bauchi—and the distribution is shown in Table 1.

Ninety-six isolates of *V. cholerae* were characterized, and all were of Ogawa serotype. All the vomitus samples grew *V*. *cholerae* O1 Ogawa; the organism was also isolated from all the water samples and the disinfectant solution collected, except in the sachet water from Gombe state (Table 2).

Fifty-four of the isolated organisms were of the *Vibrio* species other than *V.cholerae*, and those species were identified as *V. mimicus and V. parahaemolyticus.* Other organisms isolated were *Proteus* species, *Pseudomonas aeruginosa*, and *Klebsiella species* (Table 2).

**Table 1.** Type and sources of samples collected according to state

State	Rectal swab	Stool	Vomitus	Water sample					Disinfectant solution (Hypochlorite)	Total
				Borehole	Sachet	Well	Stream	Tap		
Bauchi	100	1	3	3		4	1			112
Borno	48	2	1	-	2	-	-	-	4	57
Gombe	23	2	1	-	2	2	1	1	0	32
Total	171	5	5	3	4	6	2	1	4	201

**Table 2.** Distribution of isolated organisms according to state

State	*V. cholerae* O1	*V. cholerae* non-O1	Inaba	Ogawa	Classical biotype	El Tor biotype	*V. mimicus*	*V. parahaemolyticus*	*Proteus* spp.	*P. aeruginosa*	*K. pneumonia*
Bauchi	48	32	Nil	48	48	Nil	20	6	8	4	Nil
Borno	35	20	Nil	35	35	Nil	10	10	12	6	4
Gombe	13	2	Nil	13	13	Nil	4	4	3	2	Nil
Total	96	54	Nil	96	96	Nil	34	20	23	12	4

Generally, all the isolated *Vibriocholerae* strains were sensitive to ofloxacin, gentamicin in all the states and moderately sensitive to tetracycline and doxycycline in Gombe state. Most of these were also moderately sensitive to nitrofurantoin and nalidixic acid, though not a drug of choice. The organisms were resistant to augmentin, cotrimoxazole, and amoxicillin (Table 3).

Sixty-nine (72%) of the *V. cholerae* strains were *ctx*A-positive, and none of the non-O1 strains tested was positive for *wbf*O139 gene.

**Figure 1.**
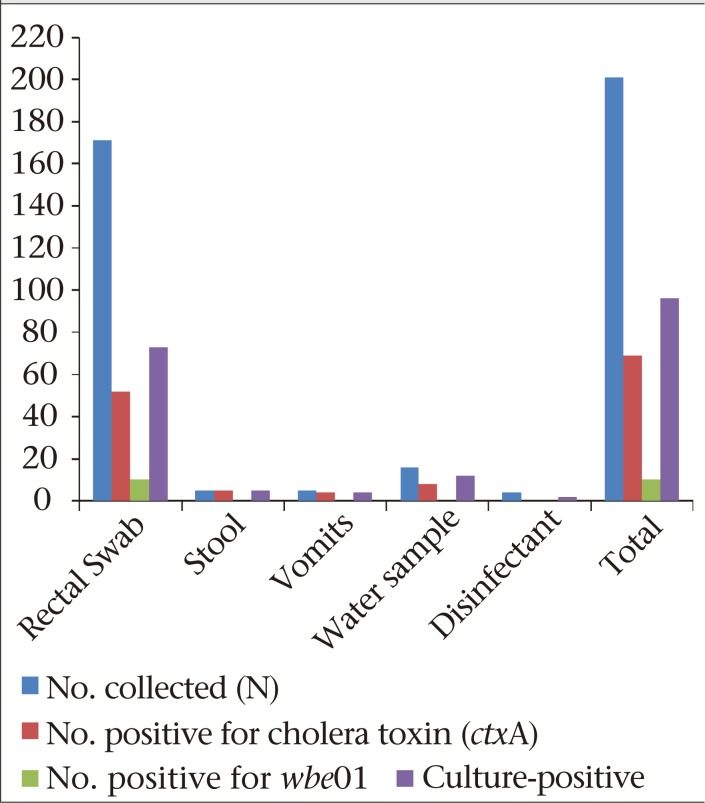
Cholera toxin (*ctx*A) and *wbe*O1 from culture-positive samples

**Figure 2.**
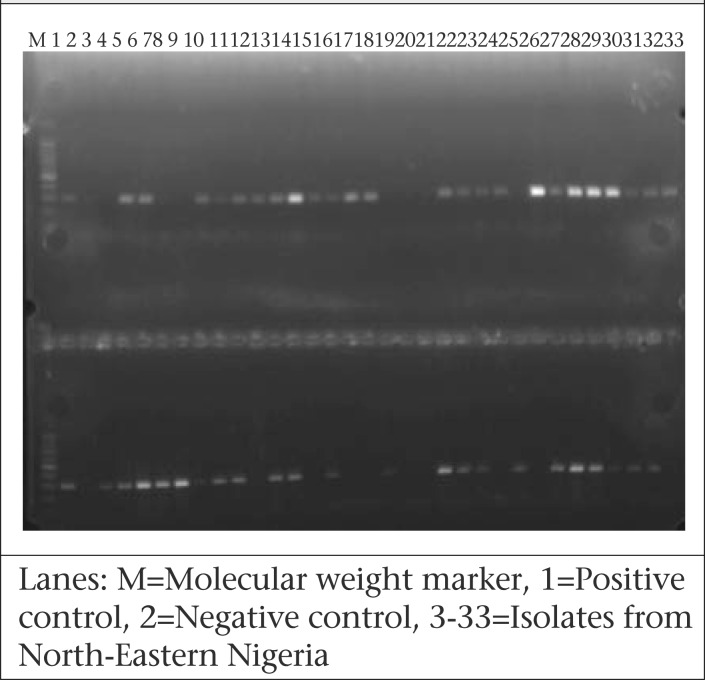
Representative agarose gel electrophoresis of *V. cholerae* O1 strains positive for *ctx*A gene, isolated during the 2010 cholera outbreak in North-Eastern Nigeria

**Figure 3.**
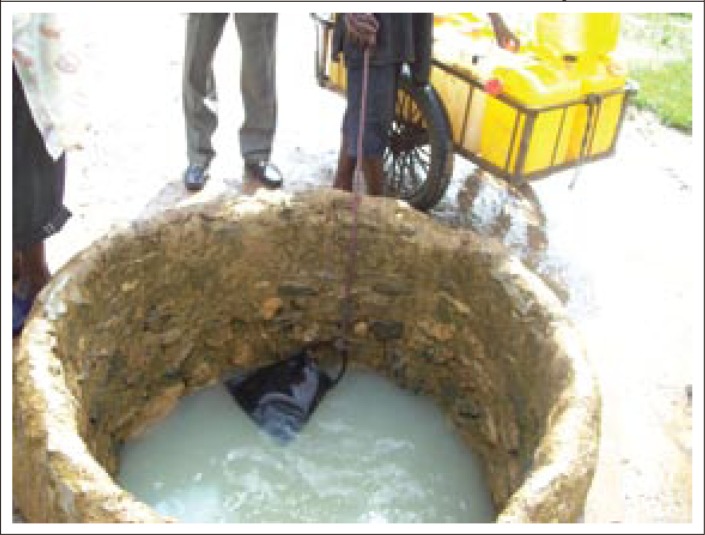
Photo of a well-water source for water vendors in the study area

Figure 1 shows the frequency of distribution of *ctx*A gene and *wbe*O1 amongst culture-positive samples while Figure 2 shows agarose gel electrophoresis of *ctx*A gene amongst *V. cholerae* isolates from North-Eastern Nigeria. Figure 3 shows one of the sources of water used by the patients that was contaminated with cholera germ.

**Table 3.** Antibiotics susceptibility patterns of the isolated *Vibrio cholerae* strains

Antibiotics	Bauchi		Borno		Gombe		Remarks
	Sensitive (%)	Resistant (%)	Sensitive (%)	Resistant (%)	Sensitive (%)	Resistant (%)	
Augmentin	18	82	8	92	32	68	Resistant
Ofloxacin	92	8	97	3	79	21	Sensitive
Gentamicin	89	11	92	8	63	37	Sensitive
Nalidixic acid	60	40	82	11	21	79	Moderate
Nitrofurantoin	78	22	85	15	68	32	Moderate
Cotrimoxazole	17	83	5	95	0	100	Resistant
Amoxicillin	0	100	0	100	26	74	Resistant
Tetracycline	89	11	93	7	58	42	Sensitive
Doxycycline	91	9	98	2	68	32	Sensitive

## DISCUSSION

The results from this study showed that the *V. cholerae* strains isolated from the 2010 outbreak in North-Eastern Nigeria were all of Ogawa serotypes and Classical biotype. This is interesting because, in the previous outbreaks in Nigeria, it had always been Ogawa serotype (15,16); however, not many reports have been published on the Classical biotype. A few reported so far have shown the El Tor biotype to predominate and affirming it as the seventh pandemic of cholera (17). In a report by Opaj *et al*. (17), the isolates were obtained from North-Central Nigeria, and these were of Inaba serotype, although some non-diarrhoeal stool was tested in this study, and these were not outbreak strains. Authors concluded, however, that *V. cholerae* was endemic in their environment. In an earlier report from the same environment in 1991, the Ogawa serotype was reported to be prevalent probably because these were outbreak strains (16).

From a recent work (18), two *V. cholerae* isolates obtained from a previous outbreak in Nigeria in 2008 (region not stated) were confirmed as Ogawa serotype and biotype El Tor, using PCR analysis of biotyping genes *tcp*A. Our study and previous studies showed that the *V. cholerae* O1 strains circulating in Nigeria included both Inaba and Ogawa, although there might be a possibility that Inaba could result from endemicity while the outbreak strains could be of Ogawa serotype. A previous study has reported that the switching of serotypes resulted from a change in the genetic make-up of the *wbt*Tgene, which de­termined the specificity of Ogawa (19).

Although the Classical biotype has been reported to be extinct, it is still present in Nigeria. There seems to be a shift between the Classical and El Tor biotype as reported in some studies where El Tor strains that produced cholera toxin of the Classical type were isolated and were, therefore, designated El Tor variants (20).

Outbreaks of cholera epidemic are usually characterized by endotoxin-producing *V. cholerae* and possibly antibiotics-resistant type. In the 2010 outbreak in Nigeria, as also observed in other outbreaks in Nigeria (1991, 1992, and 1996), the *V. cholerae* O1 were generally sensitive to ofloxacin, gentamicin, and moderately sensitive to nalidixic acid in all states, except Gombe state where resistance to tetracycline and doxycycline was as high as 42% and 32% respectively.

On the other hand, the 2010 outbreak strains of Haiti were resistant to nalidixic acid, though not the drug of choice (21). Both 2010 outbreak strains from Nigeria and Haiti were, however, resistant to cotrimoxazole, and the Nigerian strains were moderately sensitive to tetracycline.

In a previous study by Jos in Nigeria (17), all *V. cholerae* isolates were resistant to ampicillin, penicillin, chloramphenicol, and cloxacillin while these were sensitive to tetracycline (the drug of choice), ofloxacin, and erythromycin. Generally, from this study and previous ones, the isolates were sensitive to tetracycline, the drug of choice.

According to a recent report by Bhattacharya *et al*. (22), tetracycline-resistant *V. cholerae* O1 have been reported in a number of studies on major epidemics from Latin America, Tanzania, Bangladesh, and India. Resistance rates of as high as 76% were reported after a 5-month extensive use of the drug for treatment and prophylaxis.

Drug-resistant *V. cholerae* strains are, therefore, a global health concern since infections resulting from these could be more severe and difficult to treat. In addition, there could be higher case-fatality rates, prolonged hospitalizations, increased healthcare costs, and probably more secondary infections. An outbreak in Guinea-Bissau led to increased case-fatality rates from 1% to 5.3% after the outbreak strain acquired multidrug resistance (23).

It is, therefore, paramount that antimicrobials are given to patients suffering from cholera only when they have severe dehydration or other conditions that truly warrant their use. This could help reduce spread of antimicrobial drug resistance.

In addition, continuous surveillance of antimicrobial drug susceptibility patterns of *V. cholerae* should be done to lessen illness and death from the disease.

The current investigation went further than previous studies in Nigeria to confirm the production of cholera toxin by these strains, and it was found that 72% of the isolated strains had the *ctx*A gene responsible for the production of cholera toxin. However, this study has not done in-depth molecular studies to look into cholera toxin gene B (*ctx*B)-sequencing as well as screening for clonal analysis using the pulsed-field gel electrophoresis. This aspect of the work would form the next stage in the study, in which we hope to answer some of these questions concerning our outbreak strains.

None of the non-O1 *V. cholerae* strains was *V. cholerae* O139 as there was no amplification with the *wbf*O139 gene. This goes to confirm that there is currently no *V. cholerae* O139 in Nigeria, and our strains are not similar to those from Asia that have reported cases of *V. cholerae* O139 (24,25). This was also buttressed by an in-depth study compared to the Haiti outbreak strains (18).

### Conclusions

It was observed in this study that the same serotype of *V. cholerae* has been circulating during outbreaks in Nigeria, and these were of the O1 Classical biotype and Ogawa serotype. More than two-thirds of these carried the enterotoxin (*ctx*A) gene while no O139 was isolated in the study. Nevertheless, the current drug of choice proved effective but concerted efforts should be made to unravel the factors escalating the outbreak while other preventive channels, such as health education and awareness, vaccine, and clean water and sanitation, are other plausible measures.
